# Preeclampsia and risk of end stage kidney disease: A Swedish nationwide cohort study

**DOI:** 10.1371/journal.pmed.1002875

**Published:** 2019-07-30

**Authors:** Ali S. Khashan, Marie Evans, Marius Kublickas, Fergus P. McCarthy, Louise C. Kenny, Peter Stenvinkel, Tony Fitzgerald, Karolina Kublickiene

**Affiliations:** 1 School of Public Health, University College Cork, Cork, Ireland; 2 The Irish Centre for Fetal and Neonatal Translational Research (INFANT), University College Cork, Cork, Ireland; 3 Renal Medicine, Department of Clinical intervention, Science and Technology (CLINTEC), Karolinska Institutet and Karolinska University hospital, Stockholm, Sweden; 4 Department of Obstetrics and Gynaecology, Karolinska Institutet and Karolinska University hospital Stockholm, Sweden; 5 Division of Women’s Health, Women’s Health Academic Centre KHP, St. Thomas’ Hospital, London, United Kingdom; 6 Department of Women’s and Children’s Health, University of Liverpool, Liverpool, United Kingdom; 7 Department of Statistics, University College Cork, Cork, Ireland; Royal Derby Hospital, UNITED KINGDOM

## Abstract

**Background:**

Preeclampsia has been suggested to increase the risk of end-stage kidney disease (ESKD); however, most studies were unable to adjust for potential confounders including pre-existing comorbidities such as renal disease and cardiovascular disease (CVD). We aimed to examine the association between preeclampsia and the risk of ESKD in healthy women, while taking into account pre-existing comorbidity and potential confounders.

**Methods and findings:**

Using data from the Swedish Medical Birth Register (MBR), women who had singleton live births in Sweden between 1982 and 2012, including those who had preeclampsia, were identified. Women with a diagnosis of chronic kidney disease (CKD), CVD, hypertension, or diabetes prior to the first pregnancy were excluded. The outcome was a diagnosis of ESKD, identified from the Swedish Renal Registry (SRR) from January 1, 1991, onwards along with the specified cause of renal disease. We conducted Cox proportional hazards regression analysis to examine the association between preeclampsia and ESKD adjusting for several potential confounders: maternal age, body mass index (BMI), education, native country, and smoking. This analysis accounts for differential follow-up among women because women had different lengths of follow-up time. We performed subgroup analyses according to preterm preeclampsia, small for gestational age (SGA), and women who had 2 pregnancies with preeclampsia in both. The cohort consisted of 1,366,441 healthy women who had 2,665,320 singleton live births in Sweden between 1982 and 2012. At the first pregnancy, women’s mean (SD) age and BMI were 27.8 (5.13) and 23.4 (4.03), respectively, 15.2% were smokers, and 80.7% were native Swedish. The overall median (interquartile range [IQR]) follow-up was 7.4 years (3.2–17.4) and 16.4 years (10.3–22.0) among women with ESKD diagnosis. During the study period, 67,273 (4.9%) women having 74,648 (2.8% of all pregnancies) singleton live births had preeclampsia, and 410 women developed ESKD with an incidence rate of 1.85 per 100,000 person-years. There was an association between preeclampsia and ESKD in the unadjusted analysis (hazard ratio [HR] = 4.99, 95% confidence interval [CI] 3.93–6.33; *p* < 0.001), which remained in the extensively adjusted (HR = 4.96, 95% CI 3.89–6.32, *p* < 0.001) models. Women who had preterm preeclampsia (adjusted HR = 9.19; 95% CI 5.16–15.61, *p* < 0.001) and women who had preeclampsia in 2 pregnancies (adjusted HR = 7.13, 95% CI 3.12–16.31, *p* < 0.001) had the highest risk of ESKD compared with women with no preeclampsia. Considering this was an observational cohort study, and although we accounted for several potential confounders, residual confounding cannot be ruled out.

**Conclusions:**

The present findings suggest that women with preeclampsia and no major comorbidities before their first pregnancy are at a 5-fold increased risk of ESKD compared with parous women with no preeclampsia; however, the absolute risk of ESKD among women with preeclampsia remains small. Preeclampsia should be considered as an important risk factor for subsequent ESKD. Whether screening and/or preventive strategies will reduce the risk of ESKD in women with adverse pregnancy outcomes is worthy of further investigation.

## Abstract 2

Ali S Khashan and colleagues reveal an increased risk for kidney disease in women who had pre-eclampsia during pregnancy.

## Author summary

**Why was this study done?:**

- A large number of studies reported an increased risk of cardiovascular disease (CVD) among women who had preeclampsia compared with women who had no preeclampsia.
- Only few studies reported such as association between preeclampsia and the risk of developing end-stage kidney disease (ESKD).
- Some of these studies did not adjust for key potential confounders and may have lacked high-quality data.

**What did the researchers do and find?:**

- We performed this study to examine the association between preeclampsia and ESKD using a large cohort from the Swedish national registers (*N* = 1,366,441 healthy women who had 2,665,320 singleton live births).
- We found that women who had preeclampsia in at least one pregnancy, were 5 times more likely to have ESKD compared with parous women who had never had preeclampsia (hazard ratio [HR] = 4.96, 95% confidence interval [CI] 3.89–6.32).
- This association was independent of several sociodemographic factors such as maternal age and education and prepregnancy comorbidity such as renal disease and CVD.

**What do these findings mean?:**

- These results highlight the importance of preeclampsia as a risk marker for developing ESKD.
- These findings need to be investigated further to see whether preventive strategies would reduce the risk of ESKD.

## Introduction

The prevalence of chronic kidney disease (CKD) is estimated at 10% to 12% of the global population [1]. Thus, kidney disease has evolved from a subspeciality concern to a global health problem [1,2]. More recently, the scientific community has become increasingly aware of sex-specific influences on the incidence and progression of renal disorders [3]. Worldwide, it has been reported that the proportion of women with earlier stages of CKD is higher than that of men. This difference has been attributed to the longer life expectancy of women and/or potential CKD overdiagnosis due to differences in estimated glomerular filtration rate (GFR) equations [4,5]. Kidney function declines faster in men than in women, and estrogen has been suggested to play a protective role in females [6]. Estrogen also has been suggested to play a protective role in the development of cardiovascular disease (CVD) in women compared with men with end-stage kidney disease (ESKD), although this survival benefit is smaller compared with the general population [7,8]. Moreover, women’s reproductive history seems to play an important role in the subsequent risk of developing CKD. Women who have had adverse pregnancy complications may be at risk of future CKD [9,10]; however, national guidelines are still lacking the support to address the link between pregnancy history and later renal dysfunction.

Preeclampsia—defined as hypertension and the coexistence of one or more of the following new-onset conditions: proteinuria, maternal organ dysfunction (including renal insufficiency), or evidence of uteroplacental dysfunction indicated by fetal growth restriction [11,12]—complicates 2% to 8% of pregnancies and remains a major cause of maternal and perinatal mortality [13,14]. In a Norwegian study, preeclampsia has been shown to increase the future risk for ESKD [9] with the relative risk of ESKD after 1 pregnancy being 4.7 (95% confidence interval [CI] 3.6–6.1) times higher in women who had had preeclampsia versus those who had not. When the pregnancy was also complicated by low birth weight or prematurity, this further increased the risk of ESKD, suggesting that the phenotype of preeclampsia correlates with the risk of ESKD. Preeclampsia has also been shown to be associated with the subsequent development of CKD in other studies [10].

Whether the association between preeclampsia and the risk of ESKD is confounded by prepregnancy CKD, diabetes, and CVD is unknown[9], with many population cohorts lacking this critical data or else being limited by small numbers of ESKD or an inability to adjust for other relevant sociodemographic potential confounders [15,18]. Using data from the Swedish national registers, including the Swedish Renal Registry (SRR; www.snronline.se), the present population-based cohort study aimed to examine the association between preeclampsia and the risk of ESKD in healthy women without CKD, diabetes, or CVD before pregnancy.

## Methods

### Study population

Using data from the Swedish Medical Birth Register (MBR), which contains data on >99% of all births in Sweden, we identified all women who had singleton live births between January 1, 1982, and December 31, 2012. Data from the Swedish National Patient Register (NPR), the Swedish Multi-Generation Register [19], and the SRR were also used (www.snronline.se). Data from these registers were linked using the personal identification number given to all Swedish residents [20]. We excluded women who had pregnancy recorded before 1982, because data on preeclampsia before 1982 were poorly recorded. Ethical approval was obtained from Regional Ethical Review Board in Stockholm located at Karolinska Institutet.

We identified and excluded women with prior CKD, CVD, hypertension, or diabetes through a linkage with the NPR using the international classification of diseases (ICD) codes (S1 Table). Preexisting diabetes and hypertension were readily available in the MBR. This study is reported as per the Strengthening the Reporting of Observational Studies in Epidemiology (STROBE) guideline (S1 STROBE Checklist).

### Preeclampsia

Preeclampsia is recorded at the time of discharge from the hospital, using ICD-8, ICD-9, and ICD-10 (8th, 9th, and 10th revisions). Preeclampsia is defined in Sweden as a diastolic blood pressure of >90 mmHg accompanied by proteinuria (≥0.3 g/day or ≥1 on a urine dipstick). Eclampsia is defined as the occurrence of preeclampsia with seizures (S1 Table). The preeclampsia records in the MBR have previously been found to accurately reflect medical records [21].

Women who had no preeclampsia contributed follow-up time to the unexposed category from the day after the first delivery until first ESKD diagnosis, death, migration, or October 31, 2013—whichever came first. Women who had preeclampsia in the first pregnancy contributed follow-up time to the exposed category from the day after the first delivery until first ESKD diagnosis, death, migration, or October 31, 2013—whichever came first. Women who had no preeclampsia in the first pregnancy but had preeclampsia in subsequent pregnancies contributed follow-up time to the unexposed category from the first day after the first delivery until they had preeclampsia and contributed follow-up time to the exposed category from the first day after the delivery with preeclampsia until first ESKD diagnosis, death, migration, or October 31, 2013—whichever came first.

### Outcome measure

The outcome measure was ESKD. We used data from the SRR to identify women who initiated dialysis or kidney transplantation during follow-up. In addition, we used data from the Swedish Cause of Death Registry to identify women with renal disease cause of death. We used data from the SRR on all persons in Sweden who were diagnosed with ESKD from January 1, 1991, until October 31, 2013.

### Potential confounders

We had data on maternal age, number of births, body mass index (BMI), highest education level, native country, and smoking. Gestational age and small for gestational age (SGA) could have potential confounding or mediating effect on the association between preeclampsia and ESKD; therefore, they were analysed separately in stratified analyses. SGA was defined as a birth weight of 2 SDs below the mean of the sex-specific and gestational age distributions [22].

### Statistical analysis

Maternal and birth characteristics are presented according to preeclampsia status using frequency and percentages. According to a prespecified analysis plan, we used Cox proportional hazards regression to estimate the hazard ratio (HR) and 95% CIs. A Cox analysis allows the underlying risk to vary over time. It also accounts for differential follow-up time during the study period, which is very important because women had different lengths of follow-up time during the study period. The main exposure variable was any preeclampsia diagnosis in any pregnancy between 1982 and 2012 represented in the analysis as a time-dependent variable. This means that a woman with preeclampsia was considered exposed to preeclampsia from the date of delivery that was affected by preeclampsia until the end of follow-up. Considering that women who gave birth between 1982 and 1990 did not have records of ESKD until 1991 onwards, their entry was delayed until January 1991; i.e., they were not included in the analysis until registration of ESKD started in January 1991. We performed partially adjusted analyses adjusting for year of delivery. Fully adjusted models included maternal age, BMI, smoking, number of births, native country, and education. The above variables were included in the models as categorical variables, as presented in Table 1. Maternal age, BMI, smoking, and number of births were included in the models as time-dependent variables because they may potentially change across pregnancies, whereas highest education level and native country were time-fixed variables. The results of the association between each sociodemographic variable and the risk of ESKD from the main model are presented in S1 Text. The following subgroup and sensitivity analyses were prespecified.

**Table 1:** Maternal characteristics and pregnancy outcomes among healthy women at the first delivery.

Characteristic	No preeclampsia, *N* (%) *N* = 1,292,792 (95.2)	Preeclampsia, *N* (%) *N* = 65,751 (4.8)	*p-*value
**Age (years)**			*P* < 0.001
<20	60,482 (4.7)	3,272 (4.7)	
20–29	816,752 (63.2)	41,756 (63.5)	
30–39	395,997 (30.6)	19,546 (29.6)	
≥40	19,561 (1.5)	1,177 (1.8)	
**BMI in early pregnancy (kg/m**^**2**^**)**			*P* < 0.001
Underweight: <18.5	45,287 (3.5)	1,451 (2.2)	
Normal: 18.5–24.9	694,802 (53.7)	29,152 (44.3)	
Overweight: 25–29.9	183,448 (14.2)	12,801 (19.5)	
Obese: ≥30	63,195 (4.9)	7,236 (11.0)	
Missing	306,060 (23.7)	15,111 (23.0)	
**Native country**			*P* < 0.001
Sweden	1,038,664 (80.3)	56,742 (86.3)	
Other Scandinavian	39,179 (3.0)	1,932 (2.9)	
Non-Scandinavian	214,949 (16.6)	7,077 (10.8)	
**Education level**			*P* < 0.001
Pre–high school	121,962 (9.4)	5,796 (8.8)	
High school	568,755 (44.0)	31,624 (48.1)	
University level	586,564 (45.4)	27,855 (42.4)	
Missing	15,511 (1.2)	476 (0.7)	
**Maternal smoking**			*P* < 0.001
Nonsmoker	1,001,890 (77.5)	53,512 (81.4)	
0–9 cigarettes per day	135,540 (10.5)	5,367 (8.2)	
≥10 cigarettes per day	62,462 (4.8)	2,363 (3.6)	
Missing	92,900 (7.2)	4,509 (6.9)	
**Gestational diabetes**			*P* < 0.001
No	1,287,825 (99.6)	65,146 (99.1)	
Yes	4,967 (0.4)	605 (0.9)	
**Preterm birth**			*P* < 0.001
Term (≥37 weeks)	1,219,161 (94.3)	53,104 (80.8)	
Preterm (34–36 weeks)	53,566 (4.1)	7,535 (11.5)	
Very preterm (<34 weeks)	17,613 (1.4)	4,956 (7.5)	
Missing	2,452 (0.2)	156 (0.2)	
**SGA**			*P* < 0.001
No	1,244,054 (96.2)	57,224 (87.0)	
Yes	39,864 (3.1)	7,788 (11.8)	
Missing	8,874 (0.7)	739 (1.1)	
**Year of first birth**			*P* < 0.001
1982–1989	326,185 (25.2)	16,881 (25.7)	
1990–1999	409,967 (31.7)	22,236 (33.8)	
2000–2012	556,640 (43.1)	26,634 (40.5)	

A healthy woman was defined as a woman who had no recorded kidney disease, CVD, diabetes, or hypertension before the first pregnancy.

**Abbreviations**: BMI, body mass index; CVD, cardiovascular disease; SGA, small for gestational age

We analysed data separately for those women who had only 1 pregnancy and those who had 2 pregnancies only. We categorised women as follows: (1) no preeclampsia, (2) preeclampsia in 1 pregnancy, and (3) preeclampsia in both pregnancies. We plotted the Nelson-Aalen estimate of the cumulative hazard function to assess the association between preeclampsia and the risk of ESKD in the full cohort and among women who had 2 pregnancies only during the study period.

To examine the potential effect of SGA on the association between preeclampsia and ESKD, we created a 3-category variable: (1) no preeclampsia, (2) preeclampsia only, and (3) preeclampsia and SGA. Similarly, we created a 3-category variable for preterm preeclampsia: (1) no preeclampsia, (2) term preeclampsia, and (3) preterm preeclampsia. Preterm preeclampsia was defined as preeclampsia with delivery at <34 weeks’ gestation, which has been suggested in previous Swedish studies on preeclampsia [23].

Further analyses were performed by including statistical interaction terms between the preeclampsia variable and (1) maternal age (<30 versus ≥30 years), (2) BMI (overweight or obese versus normal BMI), and (3) smoking (smoker versus nonsmoker). We repeated the analysis, including women born in Sweden only, because for these women, we have access to their full reproductive history, whereas women born in other countries may have had pregnancies that are not recorded in the MBR. We repeated the analysis, excluding women who had gestational diabetes in at least 1 pregnancy. To investigate the robustness of our results, we performed several sensitivity analyses. First, we restricted the analysis to women who had their first birth in 1991 or later to ensure that our risk estimates were not biased due to lack of ESKD identification before 1991. Second, we excluded women who developed ESKD as a result of adult polycystic kidney disease (ADPKD), a hereditary renal disease not caused by preeclampsia. We explored the association between preeclampsia and cause-specific ESKD by repeating the main analysis for each primary renal disease category separately. Any missing data on a potential confounder were entered in a separate category and included in the model. We assessed the proportional hazards assumption using graphical displays of the empirical score process and found no evidence of violations of the assumption.

The population attributable risk (PAR) of ESKD is an estimate of the fraction of the total number of cases of ESKD in the population that can be attributed to a particular exposure. The estimation was carried out as described by Last [24] using the formula

$$PAR=\frac{p\times (RR-1)}{\left(1+p\times (RR-1)\right)},$$

where *p* is the proportion of the total population exposed to preeclampsia. The adjusted HR of ESKD in relation to exposure to preeclampsia was used.

Post hoc analyses were performed to (1) examine the association between preeclampsia and the risk of ESKD among women who had a diagnosis of CKD, CVD, diabetes, or hypertension before the first pregnancy (S2 Text), (2) examine the association between preeclampsia and ESKD among all women regardless of whether they had CKD, CVD, diabetes, or hypertension before the first pregnancy (S3 Text); and (3) examine the association between preeclampsia and the risk of ESKD at 5, 10, and 20 years (S4 Text).

The statistical analysis was performed in Stata version 13.1 (StataCorp, College Station, TX). All tests were two-sided with 5% significance level.

## Results

**Fig 1:**
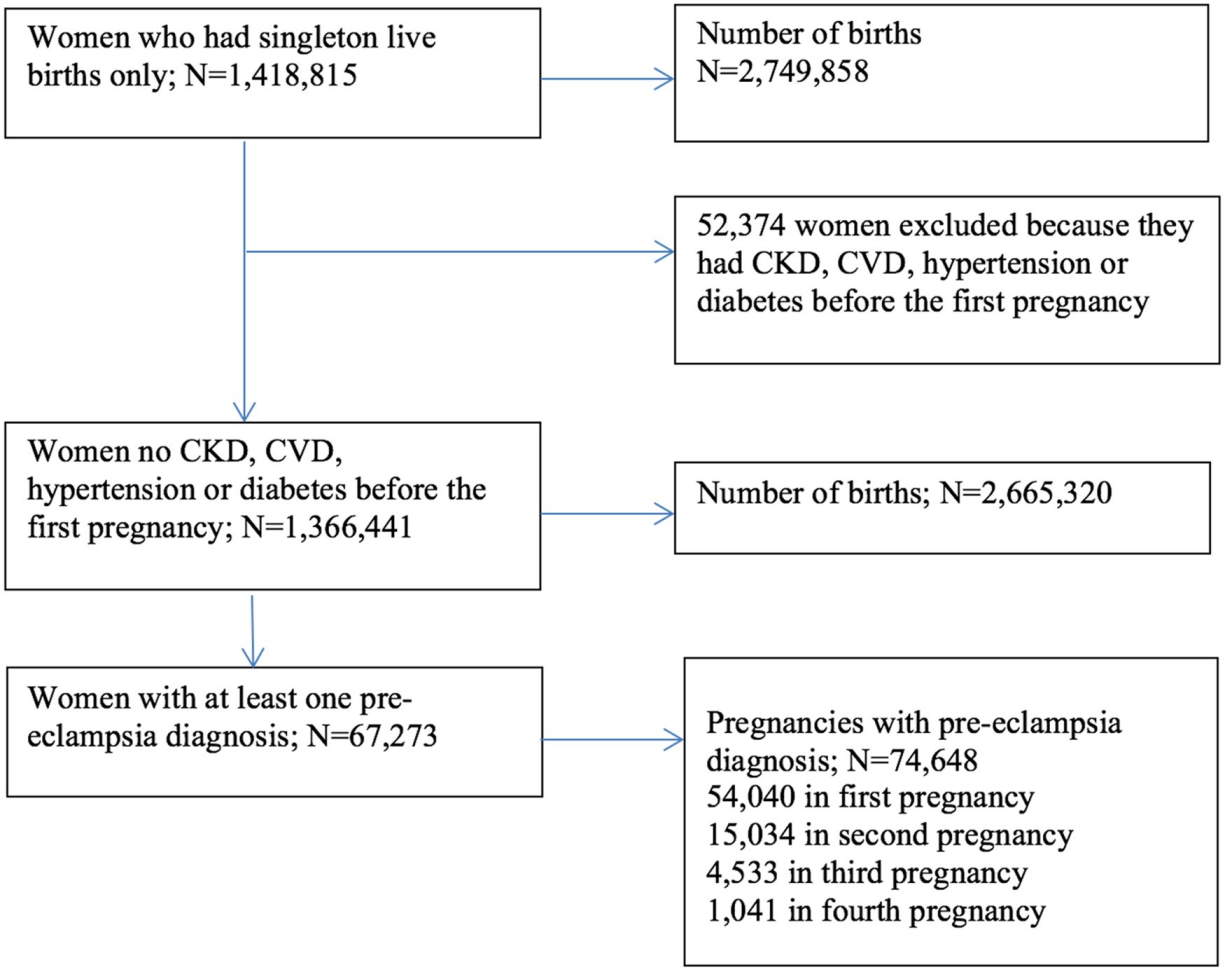
Flow chart of the study population. CKD, chronic kidney disease; CVD, cardiovascular disease.

The study cohort consisted of 1,366,441 women (2,665,320 singleton live births) with no CKD, CVD, hypertension, or diabetes before the first pregnancy. During the study period, 4.9% of women (67,273/1,366,441) had at least 1 preeclampsia diagnosis (Fig 1). ESKD was diagnosed in 410 women.

The overall median (interquartile range [IQR]) follow-up was 7.4 years (3.2–17.4) and 16.4 years (10.3–22.0) among women with ESKD diagnosis, i.e., the longer the follow-up, the more likely a woman was to develop ESKD. Women who had preeclampsia were older on average and had higher BMI. Among women with no preeclampsia in the first pregnancy, 14.2% were overweight, and 4.9% were obese compared with 20.1% and 11.8% overweight and obese, respectively, among women with preeclampsia. Women who had preeclampsia were more likely to be native Swedish (80.3% versus 86.3%) and nonsmokers (77.5% versus 81.4%). Details are summarised in Table 1.

### Preeclampsia and ESKD

**Fig 2:**
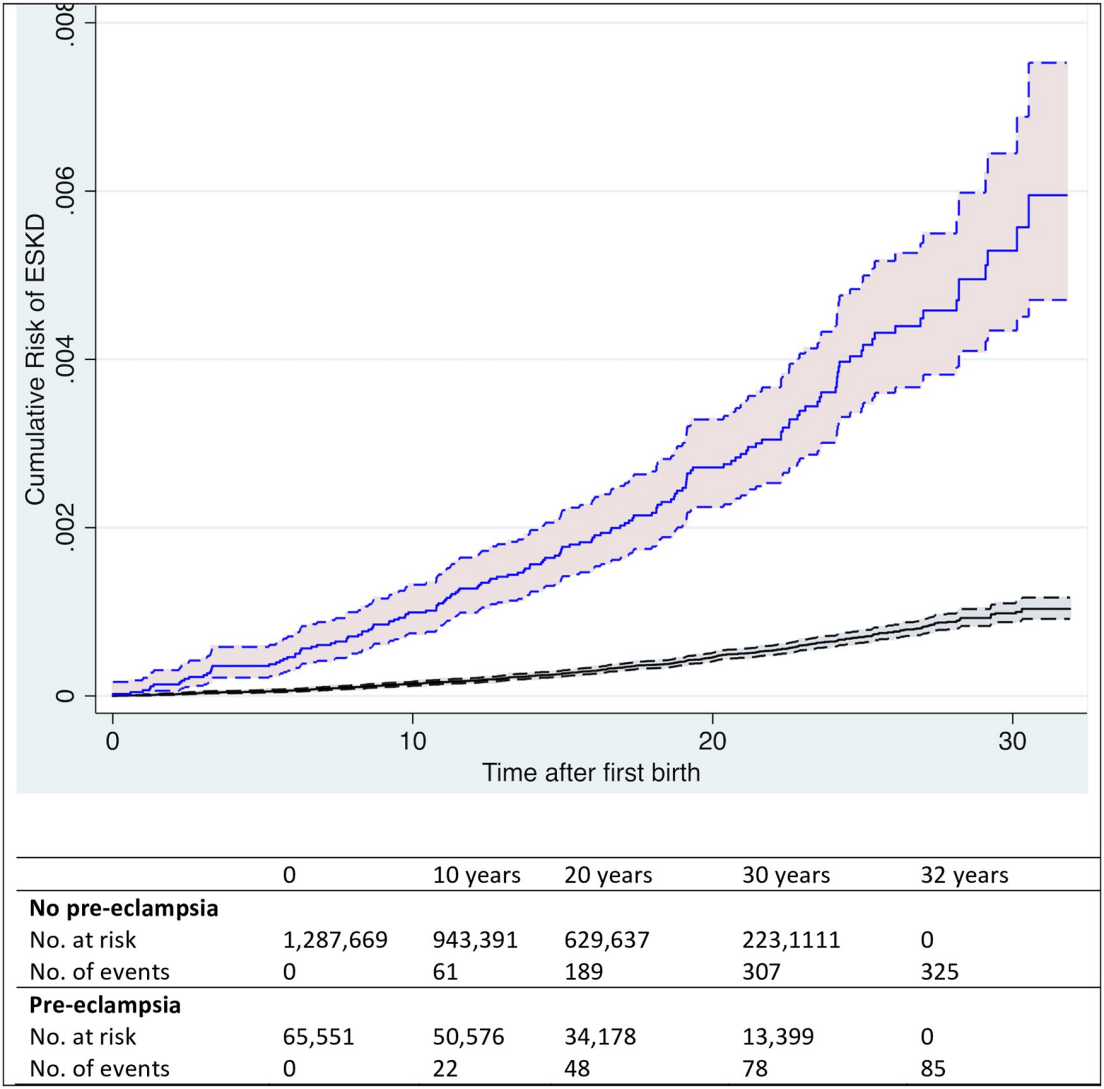
Cumulative risk of ESKD in relation to preeclampsia. The cumulative hazard plot was based on a cohort of 1,358,543 women with 2,654,641 births. Black: women with no preeclampsia; blue: women with at least one preeclampsia diagnosis; 95% CIs in dashed lines. CI, confidence interval; ESKD, end-stage kidney disease.

The incidence rate of ESKD per 100,000 person-years was 1.85 (95% CI 1.66–2.05) among women with no preeclampsia and 12.35 (95% CI 9.61–15.88) among women who had preeclampsia. There was an association between preeclampsia and the risk of end-stage renal disease (ESRD) in the crude analysis (HR = 4.99, 95% CI 3.93–6.33, *p* < 0.001), which remained largely unchanged in the adjusted model (HR = 4.96, 95% CI 3.89–6.32, *p* < 0.001). The associations between each potential confounder included in this model and the risk of ESKD are presented in S1 Text and S2 Table. When the analysis was restricted to women who had their first birth from 1991 onwards, the results supported the same conclusion, although the HR was higher (HR = 6.88; 95% CI 4.77–9.92, *p* < 0.001; Fig 2 and Table 2).

**Table 2:** HRs of the association between preeclampsia and ESKD among healthy women.

Exposure variable	Number of women with ESRD	Partially adjusted HR^a^ and 95% CI*	Adjusted HR^b^ and 95% CI*
**Entire study cohort**			
No preeclampsia	325	Reference [1]	Reference [1]
Preeclampsia	85	4.99 (3.93–6.33)	4.96 (3.89–6.32)
**Preterm preeclampsia**			
No preeclampsia	325	Reference [1]	Reference [1]
Term preeclampsia	73	4.68 (3.63–6.04)	4.67 (3.60–6.04)
Preterm preeclampsia	12	9.19 (5.16–16.35)	8.76 (4.91–15.61)
**Preeclampsia and SGA**			
No preeclampsia	325	Reference [1]	Reference [1]
Preeclampsia only	72	4.89 (3.79–6.31)	4.89 (3.77–6.34)
Preeclampsia and SGA	13	6.04 (3.47–10.51)	5.71 (3.28–9.96)
**Term preeclampsia with no SGA**			
No preeclampsia	325	Reference [1]	Reference [1]
Preeclampsia	65	4.71 (3.60–6.15)	4.73 (3.60–6.21)
**After 2 pregnancies (women with 2 pregnancies only)**			
No preeclampsia	128	Reference [1]	Reference [1]
Preeclampsia in 1 pregnancy	28	4.70 (3.12–7.08)	4.43 (2.92–6.70)
Preeclampsia in both pregnancies	6	8.24 (3.63–18.70)	7.13 (3.12–16.31)
**Entire study cohort (after 1991)**			
No preeclampsia	111	Reference [1]	Reference [1]
Preeclampsia	45	6.90 (4.83–9.86)	6.88 (4.77–9.92)
**Women with 1 pregnancy only**			
No preeclampsia	162	Reference [1]	Reference [1]
Preeclampsia	34	4.40 (3.04–6.38)	4.42 (3.03–6.46)

A healthy woman was defined as a woman who had no recorded kidney disease, CVD, diabetes, or hypertension before the first pregnancy.

*p < 0.001

^a^Model included year of delivery.

^b^Adjusted for maternal age, BMI, smoking, education, native country, year of delivery, and parity.

**Abbreviations**: CI, confidence interval; CVD, cardiovascular disease; ESKD, end-stage kidney disease; ESRD, end-stage renal disease; HR, hazard ratio; SGA, small for gestational age

### Subgroup and sensitivity analyses

**Fig 3:**
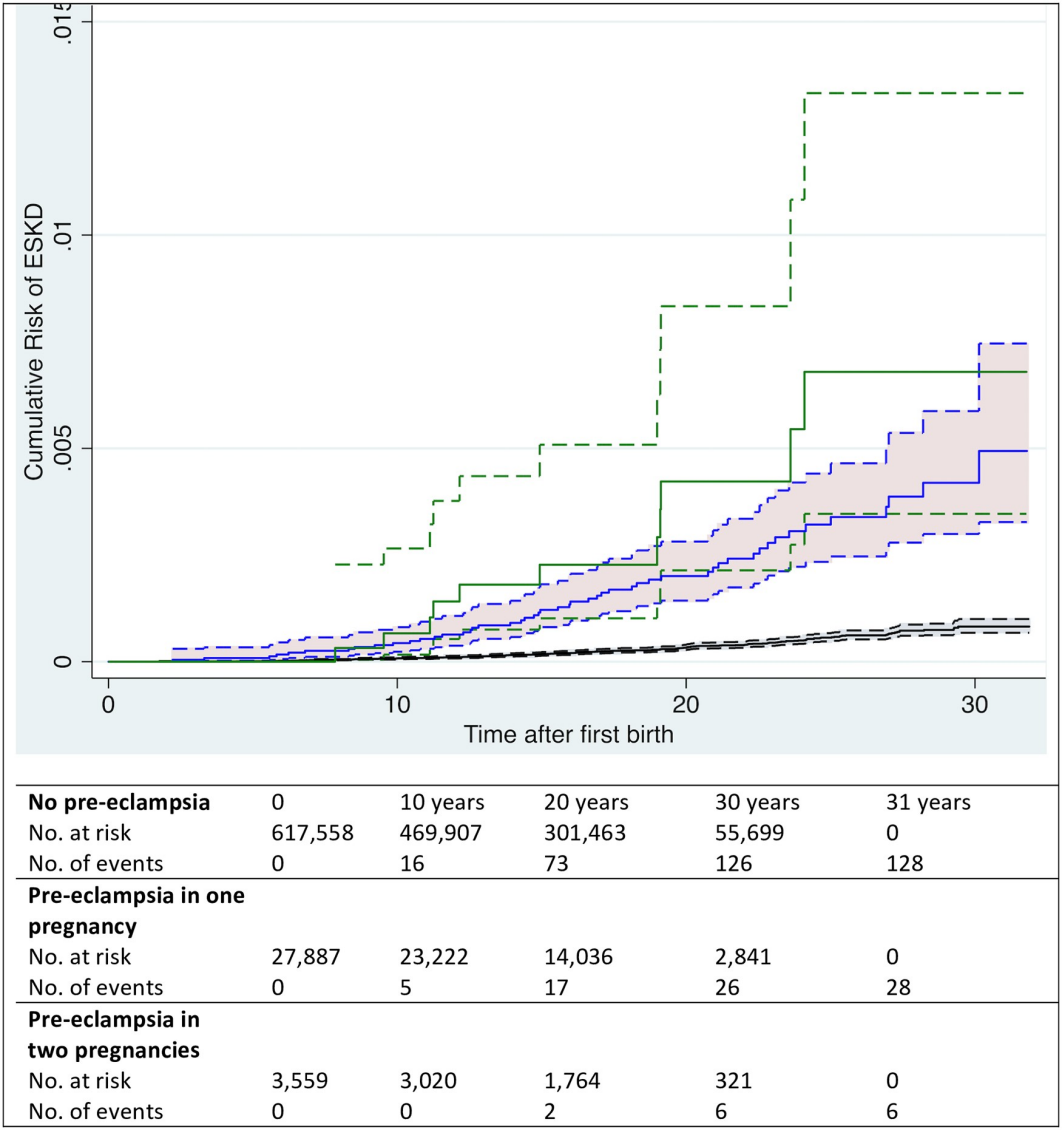
Cumulative risk of ESKD in relation to preeclampsia in 1 or 2 pregnancies among women who had 2 prepregnancies during the study period. The cumulative hazard plot was based on the cohort of 650,455 women who had 2 pregnancies recorded in the Swedish MBR during the study period; *N* = 1,291,179. Black: women with no preeclampsia; blue: women who had preeclampsia in 1 pregnancy; green: women who had preeclampsia in 2 pregnancies. 95% CIs in dashed lines. CI, confidence interval; ESKD, end-stage kidney disease; MBR, Medical Birth Register.

Women who had 2 pregnancies complicated with preeclampsia had more than 7-fold increased risk of ESKD (HR = 7.13; 95% CI 3.12–16.31, *p* < 0.001), whereas women who had preeclampsia in 1 pregnancy had a more than 4-fold increased risk of ESKD (HR = 4.43; 95% CI 2.92–6.70, *p* < 0.001; Fig 3 and Table 2). The association between term preeclampsia and ESKD was more than 4-fold (HR = 4.67; 95% CI 3.60–6.04, *p* < 0.001), whereas the association between preterm preeclampsia and the risk of ESKD was almost 9-fold (HR = 8.76; 95% CI 4.91–15.61, *p* < 0.001). The association between preeclampsia without SGA and ESKD was almost 5-fold (HR = 4.89; 95% CI 3.77–6.34, *p* < 0.001), whereas the HR was slightly larger for preeclampsia and SGA (HR = 5.71; 95% CI 3.28–9.96, *p* < 0.001).

There was no statistically significant interaction between preeclampsia and maternal age, BMI, or smoking at the first pregnancy (Table 3). Of the 410 women with ESKD in the study cohort, 96 (23.5%) were diagnosed as glomerulonephritis, 20 (4.9%) as interstitial nephritis, 51 (12.5%) as diabetic nephropathy, 16 (3.9%) as nephrosclerosis due to hypertension, 79 (19.3%) as ADPKD, 112 (27.3%) as other specified renal diseases, and 36 (8.8%) as unknown renal disease. Excluding women who developed ESKD as a result of ADPKD from the analysis resulted in a slightly smaller association (HR = 4.50; 95% CI 3.37–5.93; *p* < 0.001).

**Table 3:** The association between preeclampsia and ESKD based on subgroup analyses.

Subgroup variable	ESKD partially adjusted HR (95% CI)^a^	Interaction *p*-value	ESKD adjusted HR (95% CI)^b^	Interaction *p*-value
**Maternal age <30 years at the first pregnancy**		0.40		0.35
No preeclampsia	Reference [1]		Reference [1]	
Preeclampsia	5.57 (3.92–7.90)		5.63 (3.95–8.01)	
**Maternal age ≥30 years at the first pregnancy**				
No preeclampsia	Reference [1]		Reference [1]	
Preeclampsia	4.54 (3.27–6.29)		4.48(3.22–6.23)	
**BMI**^c^ **overweight or obese at the first pregnancy**		0.84		0.77
No preeclampsia	Reference [1]		Reference [1]	
Preeclampsia	4.51 (2.96–6.89)		4.92 (3.22–7.52)	
**Normal BMI at the first pregnancy**				
No preeclampsia	Reference [1]		Reference [1]	
Preeclampsia	4.23 (2.74–6.56)		4.50 (2.91–6.97)	
**Nonsmokers**		0.79		0.73
No preeclampsia	Reference [1]		Reference [1]	
Preeclampsia	4.95 (3.70–6.63)		4.87 (3.63–6.54)	
**Smokers**				
No preeclampsia	Reference [1]		Reference [1]	
Preeclampsia	4.61 (2.71–7.85)		4.37 (2.57–7.45)	
**Women born in Sweden**				
No preeclampsia	Reference [1]		Reference [1]	
Preeclampsia	5.20 (3.97–6.81)		4.93 (3.75–6.48)	
**Women with no gestational diabetes**				NA
No preeclampsia	Reference [1]		Reference [1]	
Preeclampsia	4.69 (3.65–6.03)		4.69 (3.63–6.05)	

^a^Model included year of delivery.

^b^Adjusted for maternal age, BMI, smoking, education, native country, year of delivery, and parity. The variable that was included in the interaction term with preeclampsia was not adjusted for. The analysis was restricted to 1 subgroup of women.

^c^The models that included interaction terms between preeclampsia and BMI excluded women with missing BMI data, and the model that included an interaction term between preeclampsia and smoking excluded women with missing smoking data.

**Abbreviations**: BMI, body mass index; CI, confidence interval; ESKD, end-stage kidney disease; HR, hazard ratio; NA, none applicable

### Preeclampsia and cause-specific ESKD

The association between preeclampsia and ESKD due to diabetes nephropathy was 9-fold (HR = 9.60; 95% CI 5.27–17.51, *p* < 0.001]) due to interstitial nephritis was 10-fold (HR = 10.54; 95% CI 4.09–27.13, *p* < 0.001), whereas it was 3-fold for glomerulonephritis (HR = 3.44; 95% CI 1.93–6.11, *p* < 0.001) and other causes (HR = 3.29; 95% CI 2.08–5.21, *p* < 0.001; Table 4). However, these results should be interpreted with caution because the number of events was small in the specific models. The numbers did not allow us to calculate the association due to hypertension.

**Table 4:** The association between preeclampsia and ESKD according to specific causes.

Cause of ESKD	Number of exposed/unexposed cases	Median follow-up (IQR) among ESKD women, years	Partially adjusted HR^a^*(95% CI)	Adjusted HR^b^*(95% CI)
Glomerulonephritis	14/82	13.87 (9.34–20.16)	3.29 (1.86–5.80)	3.44 (1.93–6.11)
Interstitial nephritis	7/13	14.27 (8.90–20.67)	10.29 (4.09–25.84)	10.54 (4.09–27.13)
Diabetic nephropathy	17/34	15.12 (8.69–23.62)	10.29 (5.73–18.46)	9.60 (5.27–17.51)
Other specified and unknown CKD	29/119	15.3 (8.46–19.62)	3.27 (2.08–5.14)	3.29 (2.08–5.21)

^a^Model included year of delivery.

^b^Adjusted for maternal age, BMI, smoking, education, native country, year of delivery, and parity.

*All *p* < 0.001

**Abbreviations**: BMI, body mass index; CI, confidence interval; CKD, chronic kidney disease; ESKD, end-stage kidney disease; HR, hazard ratio; IQR, interquartile range

### PAR

The proportion of women who had preeclampsia in the cohort was 0.048, and the adjusted HR was 4.50 (excluding women who had ESKD caused by ADPKD); therefore, the population attributable fraction associated with preeclampsia was 0.14. Thus, in our study, exposure to preeclampsia accounted for 14% of all ESKD in the studied population.

### Preeclampsia and ESKD among women with prepregnancy comorbidity and among all women

The results of the association between preeclampsia and ESKD among women who had CKD, CVD, hypertension, or diabetes before the first pregnancy (S2 Text and S3 Table) and among all women combined (S3 Text and S4 Table) are presented in Supporting information. The associations between preeclampsia and the risk of ESKD at 5, 10, and 20 years are also presented in Supporting information (S4 Text and S5 Table).

## Discussion

This large, nationwide cohort study of healthy women followed up to 30 years after their first pregnancy suggests that women who have preeclampsia are at almost 5-fold increased risk to develop ESKD. The association is independent of a number of key potential confounders, including pre-existing CKD, CVD, diabetes, and hypertension. The highest risk of ESKD was observed among women with preterm preeclampsia, preeclampsia and SGA in the same pregnancy, and women who had preeclampsia in 2 pregnancies. The highest risk was associated with development of diabetic nephropathy. The population attributable fraction suggested that preeclampsia was responsible for 14% of all ESKD cases in parous women, although this estimate is based on the assumption that the association between preeclampsia and ESKD is causal. Restricting the analysis to women who had their first delivery from 1991 onwards resulted in a higher HR. Our results suggested that the risk of ESKD was increased in relation to preeclampsia in the first 5 years and the first 10-year follow-up (S5 Table), which may suggest that the increased HR could be due to missed ESKD diagnoses between 1982 and 1990 and that the true HR is likely to be larger than 5.

The present findings are consistent with a population-based study from Norway, which included about 570,000 women compared with our 1.36 million [9]. However, we were unable to compare the HR estimates on healthy women from our study with the Norwegian study because they did not report the results when they excluded women with prepregnancy comorbidity. Our results are consistent with the results of the Norwegian study in that the association between preeclampsia would remain statistically significant whether the women with prepregnancy comorbidity are included or excluded. In addition, the larger cohort in the present study allowed us to perform robust subgroup analyses.

Two other studies using insurance claims data on approximately 240,000 and 940,000 women, respectively, both from Taiwan, reported an increased risk of ESKD in relation to hypertensive disorders in pregnancy, including gestational hypertension and preeclampsia [17,25]. The reported results for preeclampsia ranged from an HR of 2 to 15 depending on the analytical approach; however, neither of the studies excluded women with prepregnancy CKD, CVD, hypertension, and diabetes. Another recent nested case-control study within the United States Renal Data System included 44 women with ESKD, and 88 controls [15] reported a 3-fold increased risk of ESKD in relation to preeclampsia. Our findings are also consistent with a recent study that used a large data set from the Canada Institute for Health Information [26]. Although the statistical analysis was not adjusted for several potential confounding factors such as maternal education, BMI, smoking, and prepregnancy comorbidity, the findings on preeclampsia and ESKD are consistent with our findings. A recent study found that none of the women within the Prevention of Renal and Vascular Endstage Disease Study (PREVEND) cohort developed ESKD during follow-up; however, this is not surprising considering that the study cohort consisted of less than 3,000 women [27].

The precise pathophysiological mechanisms linking preeclampsia and ESKD are not clear. CKD and preeclampsia have common risk factors (e.g., obesity, hypertension, and insulin resistance), indicating a shared aetiology most likely through the widespread endothelial dysfunction that has been shown to be present in both preeclampsia and ESKD [28]. Similarly, preeclampsia is characterised by the pathognomonic lesion of glomerular endotheliosis [29], and there is also evidence of primary renal injury via podocyte loss that fails to fully recover following pregnancy in some women and may result in this increased risk [30,31]. In our study, we observed that preeclampsia was associated with a 9-fold increased risk of ESKD from diabetes nephropathy, even though women with prior diabetes were excluded and the models accounted for gestational diabetes and BMI. It is possible that a certain genotype could be the common link between development of preeclampsia and CKD or even modify the risk of developing ESKD in those who later develop diabetes [32,33]. Indeed, abnormal expression and signalling of the transcription factor nuclear factor erythroid 2(NF-E2)-related factor 2-Kelch-like erythroid cell-derived protein with CNC homology (ESH)-associated protein 1 (Nrf2-KEAP1) has been associated with the development of preeclampsia [34]. It could be speculated that preeclampsia reflects a susceptibility to develop burden of lifestyle diseases within the Nrf2 diseasome, such as CKD, diabetes, and CVD [35].

Previous studies have shown that women with a history of preeclampsia have an increased incidence of microalbuminuria, a marker for renal disease [36] and predictor of adverse renal outcomes [37]. This suggests either that these women might have underlying previously unrecognised renal disease, which is unmasked by preeclampsia, or that preeclampsia adversely affects long-term renal function resulting in an increased risk of developing CKD. After a pregnancy complicated by preeclampsia, the renal disturbances usually normalise but not necessarily to baseline levels, and the presence of albuminuria and changes in renal haemodynamics have been observed several years after the delivery. A meta-analysis showed that the occurrence of micro-albuminuria is as high as 31% in women with previous preeclampsia compared with 7% in controls at a weighted mean of 7.1 years post partum [16]. In addition, they reported a graded relationship between microalbuminuria and the severity of preeclampsia, with a 4-fold increase after mild preeclampsia and an 8-fold increase after preterm preeclampsia. Because other studies reported that 20% to 40% of women with preeclampsia have microalbuminuria 3 to 5 years after pregnancy compared with only 2% of women without preeclampsia [36,38], this may be interpreted as supporting a causal association.

Changes in renal haemodynamics postpartum in women with preeclampsia have also been suggested to contribute to an increased risk of ESKD. However, the few studies that assessed renal function and haemodynamics after preeclamptic pregnancy [16] suggested only small changes in renal function. Changes in renal haemodynamics are believed to be primarily related to the increased estimated glomerular filtration rate (eGFR) towards the hyperfiltration state in addition to a presence of increased filtration fraction [39,40]. Because the changes are more pronounced in women with history of preterm preeclampsia, this could support the link between preeclampsia and CKD. It has been suggested that hyperfiltration might serve as an initial sign for renal abnormalities, in which altered filtration fraction could contribute to progressive renal damage and decline in GFR [41,42].

### Strengths and limitations

Our study has several strengths. First, it is the largest study on preeclampsia and ESKD to date using one of the best available epidemiological data sources worldwide. The MBR contains data on more than 99% of all births in Sweden, which rules out selection bias due to nonparticipation. Furthermore, the data were collected prospectively, and the preeclampsia diagnoses were ascertained by obstetricians and registered during follow-up in the MBR, which minimised the risk of misclassification. Linkage to the national and validated SRR confirmed the ESKD diagnosis directly and reduced the risk of misclassification of outcome commonly seen when using only administrative databases. We were able to identify the primary renal diagnosis made by the nephrologist. Our access to data on maternal comorbidity before the first pregnancy and during the reproductive period is a significant strength, which allowed us to address the potential confounding of CKD, diabetes, hypertension, and CVD by excluding them from the main analysis. In addition, due to the prospectively collected information in the MBR, we were able to adjust for key sociodemographic variables, including age, BMI, smoking, education, and native country. The size of the cohort allowed us to perform several subgroup and sensitivity analyses.

The study has some limitations that should be considered when interpreting the results. Although we excluded women who had a recorded prepregnancy diagnosis of a major related comorbidity, we cannot rule out that some women may have had undiagnosed or unknown disease leading to residual confounding. Although the study is population based, the results are not generalisable to women who had stillbirth or twins because these were not included in the cohort. However, the percentage of women who may have had stillbirth or twins is very small. Also, we did not have data on miscarriages during the study period; however, this is unlikely to influence the reported results because these women would not have had preeclampsia. We cannot rule out potential confounding by environmental factors related to diet and physical activity and genetic factors, which we did not have access to. Finally, data on BMI were missing in 23% of the population; however, this was similar between exposed and unexposed groups, and maternal characteristics did not appear to confound the association between preeclampsia and ESKD.

### Conclusion

We report that healthy women with preeclampsia are at a 5-fold increased risk of ESKD compared with parous women with no preeclampsia. This shows that preeclampsia is a sex-specific, independent risk factor for the subsequent development of ESKD. However, it should be noted that the overall ESKD risk remains small. Whether screening and/or preventative strategies will reduce the risk of ESKD in women with adverse pregnancy outcomes is worthy of further investigation.

## Supporting information

S1 STROBE Checklist (DOC)

S1 Text The HRs and 95% CIs of the sociodemographic factors and the risk of ESKD. CI, confidence interval; ESKD, end-stage kidney disease; HR, hazard ratio.
(DOCX)

S2 Text The association between preeclampsia and ESKD among women with prepregnancy comorbidity. ESKD, end-stage kidney disease.
(DOCX)

S3 Text The association between preeclampsia and ESKD among all women regardless of prepregnancy comorbidity. ESKD, end-stage kidney disease.
(DOCX)

S4 Text The association between preeclampsia and ESKD at 5-, 10-, and 20-year follow-up. ESKD, end-stage kidney disease.
(DOCX)

S1 Table ICD codes. ICD, international classification of diseases.
(XLSX)

S2 Table The HRs of the association between each potential confounder, included in the main analysis, and ESKD among healthy women. ESKD, end-stage kidney disease; HR, hazard ratio.
(XLSX)

S3 Table HRs of the association between preeclampsia and ESKD among women with prepregnancy comorbidity. ESKD, end-stage kidney disease; HR, hazard ratio.
(XLSX)

S4 Table HRs of the association between preeclampsia and ESKD among all women regardless of prepregnancy medical history. ESKD, end-stage kidney disease; HR, hazard ratio.
(XLSX)

S5 Table The HRs of the association between preeclampsia and ESKD among healthy women, women with prepregnancy comorbidity, and all women at 5-, 10-, and 20-year follow-up. All HRs are based on comparing women who had preeclampsia in at least 1 pregnancy with women who never had preeclampsia during the follow-up period. ESKD, end-stage kidney disease; HR, hazard ratio.
(XLSX)

## Abbreviations

ADPKD: adult polycystic kidney disease
BMI: body mass index
CI: confidence interval
CKD: chronic kidney disease
CVD: cardiovascular disease
eGFR: estimated glomerular filtration rate
ESKD: end-stage kidney disease
ESRD: end-stage renal disease
GFR: glomerular filtration rate
HR: hazard ratio
ICD: international classification of diseases
IQR: interquartile range
KEAP1: Kelch-like erythroid cell-derived protein with CNC homology (ESH)-associated protein 1
MBR: Medical Birth Register
NPR: National Patient Register
Nrf2: nuclear factor erythroid 2(NF-E2)-related factor 2
PAR: population attributable risk
PREVEND: Prevention of Renal and Vascular Endstage Disease Study
SGA: small for gestational age
SRR: Swedish Renal Registry
STROBE: Strengthening the Reporting of Observational Studies in Epidemiology

## References

[1] Levin A, Tonelli M, Bonventre J, Coresh J, Donner JA, Fogo AB, et al Global kidney health 2017 and beyond: a roadmap for closing gaps in care, research, and policy. Lancet (London, England). 2017;390(10105):1888–917. Epub 2017/04/25. 10.1016/s0140-6736(17)30788-2 . 28434650

[2] Stenvinkel P. Chronic kidney disease: a public health priority and harbinger of premature cardiovascular disease. Journal of internal medicine. 2010;268(5):456–67. Epub 2010/09/03. 10.1111/j.1365-2796.2010.02269.x . 20809922

[3] Ashuntantang GE, Garovic VD, Heilberg IP, Lightstone L. Kidneys and women’s health: key challenges and considerations. Nature reviews Nephrology. 2018;14(3):203–10. Epub 2018/01/31. 10.1038/nrneph.2017.188 . 29380816

[4] Mills KT, Xu Y, Zhang W, Bundy JD, Chen CS, Kelly TN, et al A systematic analysis of worldwide population-based data on the global burden of chronic kidney disease in 2010. Kidney international. 2015;88(5):950–7. Epub 2015/07/30. 10.1038/ki.2015.230 . 26221752 PMC4653075

[5] Cobo G, Hecking M, Port FK, Exner I, Lindholm B, Stenvinkel P, et al Sex and gender differences in chronic kidney disease: progression to end-stage renal disease and haemodialysis. Clinical science (London, England: 1979). 2016;130(14):1147–63. Epub 2016/06/03. 10.1042/cs20160047 . 27252402

[6] Silbiger SR, Neugarten J. The role of gender in the progression of renal disease. Advances in renal replacement therapy. 2003;10(1):3–14. Epub 2003/03/05. 10.1053/jarr.2003.50001 . 12616458

[7] Carrero JJ, de Jager DJ, Verduijn M, Ravani P, De Meester J, Heaf JG, et al Cardiovascular and noncardiovascular mortality among men and women starting dialysis. Clinical journal of the American Society of Nephrology: CJASN. 2011;6(7):1722–30. Epub 2011/07/08. 10.2215/CJN.11331210 . 21734088

[8] Carrero JJ, Hecking M, Chesnaye NC, Jager KJ. Sex and gender disparities in the epidemiology and outcomes of chronic kidney disease. Nature reviews Nephrology. 2018;14(3):151–64. Epub 2018/01/23. 10.1038/nrneph.2017.181 . 29355169

[9] Vikse BE, Irgens LM, Leivestad T, Skjaerven R, Iversen BM. Preeclampsia and the risk of end-stage renal disease. The New England journal of medicine. 2008;359(8):800–9. 10.1056/NEJMoa0706790 . 18716297

[10] Vikse BE, Irgens LM, Karumanchi SA, Thadhani R, Reisaeter AV, Skjaerven R. Familial factors in the association between preeclampsia and later ESRD. Clinical journal of the American Society of Nephrology: CJASN. 2012;7(11):1819–26. 10.2215/CJN.01820212 . 22956264 PMC3488941

[11] Tranquilli AL, Dekker G, Magee L, Roberts J, Sibai BM, Steyn W, et al The classification, diagnosis and management of the hypertensive disorders of pregnancy: A revised statement from the ISSHP. Pregnancy hypertension. 2014;4(2):97–104. 10.1016/j.preghy.2014.02.001 . 26104417

[12] Brown MA, Magee LA, Kenny LC, Karumanchi SA, McCarthy FP, Saito S, et al The hypertensive disorders of pregnancy: ISSHP classification, diagnosis & management recommendations for international practice. Pregnancy hypertension. 2018 Epub 2018/05/29. 10.1016/j.preghy.2018.05.004 . 29803330

[13] Wildman K, Bouvier-Colle MH. Maternal mortality as an indicator of obstetric care in Europe. Bjog. 2004;111(2):164–9. Epub 2004/01/16. . 14723755 10.1046/j.1471-0528.2003.00034.x-i1

[14] Souza JP, Gulmezoglu AM, Vogel J, Carroli G, Lumbiganon P, Qureshi Z, et al Moving beyond essential interventions for reduction of maternal mortality (the WHO Multicountry Survey on Maternal and Newborn Health): a cross-sectional study. Lancet. 2013;381(9879):1747–55. 10.1016/S0140-6736(13)60686-8 . 23683641

[15] Kattah AG, Scantlebury DC, Agarwal S, Mielke MM, Rocca WA, Weaver AL, et al Preeclampsia and ESRD: The Role of Shared Risk Factors. American journal of kidney diseases: the official journal of the National Kidney Foundation. 2017;69(4):498–505. 10.1053/j.ajkd.2016.07.034 . 27707553 PMC5366077

[16] McDonald SD, Han Z, Walsh MW, Gerstein HC, Devereaux PJ. Kidney disease after preeclampsia: a systematic review and meta-analysis. American journal of kidney diseases: the official journal of the National Kidney Foundation. 2010;55(6):1026–39. 10.1053/j.ajkd.2009.12.036 . 20346562

[17] Wang IK, Muo CH, Chang YC, Liang CC, Chang CT, Lin SY, et al Association between hypertensive disorders during pregnancy and end-stage renal disease: a population-based study. CMAJ: Canadian Medical Association journal = journal de l’Association medicale canadienne. 2013;185(3):207–13. 10.1503/cmaj.120230 . 23339156 PMC3576438

[18] Mannisto T, Mendola P, Vaarasmaki M, Jarvelin MR, Hartikainen AL, Pouta A, et al Elevated blood pressure in pregnancy and subsequent chronic disease risk. Circulation. 2013;127(6):681–90. 10.1161/CIRCULATIONAHA.112.128751 . 23401113 PMC4151554

[19] Ludvigsson JF, Almqvist C, Bonamy AK, Ljung R, Michaelsson K, Neovius M, et al Registers of the Swedish total population and their use in medical research. European journal of epidemiology. 2016;31(2):125–36. Epub 2016/01/16. 10.1007/s10654-016-0117-y . 26769609

[20] Ludvigsson JF, Otterblad-Olausson P, Pettersson BU, Ekbom A. The Swedish personal identity number: possibilities and pitfalls in healthcare and medical research. European journal of epidemiology. 2009;24(11):659–67. Epub 2009/06/09. 10.1007/s10654-009-9350-y . 19504049 PMC2773709

[21] Ros HS, Cnattingius S, Lipworth L. Comparison of risk factors for preeclampsia and gestational hypertension in a population-based cohort study. American journal of epidemiology. 1998;147(11):1062–70. Epub 1998/06/10. 10.1093/oxfordjournals.aje.a009400 . 9620050

[22] Marsal K, Persson PH, Larsen T, Lilja H, Selbing A, Sultan B. Intrauterine growth curves based on ultrasonically estimated foetal weights. Acta paediatrica (Oslo, Norway: 1992). 1996;85(7):843–8. Epub 1996/07/01. . 8819552 10.1111/j.1651-2227.1996.tb14164.x

[23] Hernandez-Diaz S, Toh S, Cnattingius S. Risk of pre-eclampsia in first and subsequent pregnancies: prospective cohort study. Bmj. 2009;338:b2255 Epub 2009/06/23. 10.1136/bmj.b2255 . 19541696 PMC3269902

[24] Last J. A Dictionary of Epidemiology. Porta M, editor. Oxford, England, Oxford University Press; 2001.

[25] Wu CC, Chen SH, Ho CH, Liang FW, Chu CC, Wang HY, et al End-stage renal disease after hypertensive disorders in pregnancy. American journal of obstetrics and gynecology. 2014;210(2):147 e1–8. 10.1016/j.ajog.2013.09.027 . 24060448

[26] Dai L, Chen Y, Sun W, Liu S. Association Between Hypertensive Disorders During Pregnancy and the Subsequent Risk of End-Stage Renal Disease: A Population-Based Follow-Up Study. Journal of obstetrics and gynaecology Canada: JOGC = Journal d’obstetrique et gynecologie du Canada: JOGC. 2018;40(9):1129–38. Epub 2018/06/24. 10.1016/j.jogc.2018.01.022 . 29934233

[27] Paauw ND, van der Graaf AM, Bozoglan R, van der Ham DP, Navis G, Gansevoort RT, et al Kidney Function After a Hypertensive Disorder of Pregnancy: A Longitudinal Study. American journal of kidney diseases: the official journal of the National Kidney Foundation. 2018;71(5):619–26. Epub 2018/01/01. 10.1053/j.ajkd.2017.10.014 . 29289477

[28] Steegers EA, von Dadelszen P, Duvekot JJ, Pijnenborg R. Pre-eclampsia. Lancet (London, England). 2010;376(9741):631–44. 10.1016/S0140-6736(10)60279-6 . 20598363

[29] Wagner SJ, Craici IM, Grande JP, Garovic VD. From placenta to podocyte: vascular and podocyte pathophysiology in preeclampsia. Clin Nephrol. 2012;78(3):241–9. 10.5414/cn107321 . 22874114

[30] White WM, Garrett AT, Craici IM, Wagner SJ, Fitz-Gibbon PD, Butters KA, et al Persistent urinary podocyte loss following preeclampsia may reflect subclinical renal injury. PLoS ONE. 2014;9(3):e92693 10.1371/journal.pone.0092693 . 24664365 PMC3963957

[31] Karumanchi SA, Maynard SE, Stillman IE, Epstein FH, Sukhatme VP. Preeclampsia: a renal perspective. Kidney international. 2005;67(6):2101–13. 10.1111/j.1523-1755.2005.00316.x . 15882253

[32] Sowmya S, Ramaiah A, Nallari P, Jyothy A, Venkateshwari A. Role of IL-6–174(G/C) promoter polymorphism in the etiology of early-onset preeclampsia. Inflammation research: official journal of the European Histamine Research Society [et al]. 2015;64(6):433–9. Epub 2015/04/29. 10.1007/s00011-015-0823-z . 25917045

[33] Spoto B, Mattace-Raso F, Sijbrands E, Leonardis D, Testa A, Pisano A, et al Association of IL-6 and a functional polymorphism in the IL-6 gene with cardiovascular events in patients with CKD. Clinical journal of the American Society of Nephrology: CJASN. 2015;10(2):232–40. Epub 2014/12/11. 10.2215/CJN.07000714 . 25492254 PMC4317745

[34] Kweider N, Huppertz B, Kadyrov M, Rath W, Pufe T, Wruck CJ. A possible protective role of Nrf2 in preeclampsia. Annals of anatomy = Anatomischer Anzeiger: official organ of the Anatomische Gesellschaft. 2014;196(5):268–77. Epub 2014/06/24. 10.1016/j.aanat.2014.04.002 . 24954650

[35] Cuadrado A, Manda G, Hassan A, Alcaraz MJ, Barbas C, Daiber A, et al Transcription Factor NRF2 as a Therapeutic Target for Chronic Diseases: A Systems Medicine Approach. Pharmacological reviews. 2018;70(2):348–83. Epub 2018/03/07. 10.1124/pr.117.014753 . 29507103

[36] Bar J, Kaplan B, Wittenberg C, Erman A, Boner G, Ben-Rafael Z, et al Microalbuminuria after pregnancy complicated by pre-eclampsia. Nephrology, dialysis, transplantation: official publication of the European Dialysis and Transplant Association—European Renal Association. 1999;14(5):1129–32. Epub 1999/05/27. 10.1093/ndt/14.5.1129 . 10344350

[37] Gerstein HC, Mann JF, Yi Q, Zinman B, Dinneen SF, Hoogwerf B, et al Albuminuria and risk of cardiovascular events, death, and heart failure in diabetic and nondiabetic individuals. JAMA. 2001;286(4):421–6. 10.1001/jama.286.4.421 . 11466120

[38] Nisell H, Lintu H, Lunell NO, Mollerstrom G, Pettersson E. Blood pressure and renal function seven years after pregnancy complicated by hypertension. British journal of obstetrics and gynaecology. 1995;102(11):876–81. Epub 1995/11/01. . 8534622 10.1111/j.1471-0528.1995.tb10874.x

[39] Sandvik MK, Hallan S, Svarstad E, Vikse BE. Preeclampsia and prevalence of microalbuminuria 10 years later. Clinical journal of the American Society of Nephrology: CJASN. 2013;8(7):1126–34. Epub 2013/06/01. 10.2215/CJN.10641012 . 23723340 PMC3700700

[40] Toering TJ, van der Graaf AM, Visser FW, Groen H, Faas MM, Navis G, et al Higher filtration fraction in formerly early-onset preeclamptic women without comorbidity. American journal of physiology Renal physiology. 2015;308(8):F824–31. Epub 2015/02/20. 10.1152/ajprenal.00536.2014 . 25694481

[41] Helal I, Fick-Brosnahan GM, Reed-Gitomer B, Schrier RW. Glomerular hyperfiltration: definitions, mechanisms and clinical implications. Nature reviews Nephrology. 2012;8(5):293–300. Epub 2012/02/22. 10.1038/nrneph.2012.19 . 22349487

[42] Palatini P, Mormino P, Dorigatti F, Santonastaso M, Mos L, De Toni R, et al Glomerular hyperfiltration predicts the development of microalbuminuria in stage 1 hypertension: the HARVEST. Kidney international. 2006;70(3):578–84. Epub 2006/06/22. 10.1038/sj.ki.5001603 . 16788693

