# Correction: Preeclampsia and risk of end stage kidney disease: A Swedish nationwide cohort study

**DOI:** 10.1371/journal.pmed.1002977

**Published:** 2019-10-24

**Authors:** Ali S. Khashan, Marie Evans, Marius Kublickas, Fergus P. McCarthy, Louise C. Kenny, Peter Stenvinkel, Tony Fitzgerald, Karolina Kublickiene

In the Funding section, the following information was inadvertently omitted: Additional funding to KK from the Swedish Research Council grant no. 2018–00932. The full funding statement should read:

The study was partly funded by the Irish Centre for Fetal and Neonatal Translational Research (INFANT) (grant no. 12/RC/2272), Strategic Research Programme in Diabetes at Karolinska Institutet (Swedish Research Council grant No 2009–1068), the Stockholm County Council (ALF), and the Swedish Kidney Foundation. Additional funding to KK from the Swedish Research Council grant no. 2018–00932. No funding bodies had any role in study design, data collection and analysis, decision to publish, or preparation of the manuscript.
