# Supplementary material for: Preeclampsia and risk of end stage kidney disease: A Swedish nationwide cohort study
Source: PLoS Med. 2019 Jul 30;16(7):e1002875. doi: 10.1371/journal.pmed.1002875 (PMC6667103; doi:10.1371/journal.pmed.1002875)
Supplement: S1 STROBE Checklist — (DOC) [file pmed.1002875.s001.doc]

STROBE Statement—Checklist of items that should be included in reports of ***cohort studies***

|  | Item No | Recommendation | Paragraph and page number |
| --- | --- | --- | --- |
| **Title and abstract** | 1 | (*a*) Indicate the study’s design with a commonly used term in the title or the abstract | Title and Methods and Findings section |
|  |  | (*b*) Provide in the abstract an informative and balanced summary of what was done and what was found | Methods and Findings section |
| Introduction | | |  |
| Background/rationale | 2 | Explain the scientific background and rationale for the investigation being reported | Paragraphs 1 and 2 of Introduction |
| Objectives | 3 | State specific objectives, including any prespecified hypotheses | Paragraph 1 of Introduction |
| Methods | | |  |
| Study design | 4 | Present key elements of study design early in the paper | First and second paragraphs of the Methods section |
| Setting | 5 | Describe the setting, locations, and relevant dates, including periods of recruitment, exposure, follow-up, and data collection | First and second paragraphs of the Methods section |
| Participants | 6 | (*a*) Give the eligibility criteria, and the sources and methods of selection of participants. Describe methods of follow-up | First and second paragraphs of the Methods section. Follow-up is described in the fourth paragraph of the Methods section. |
|  |  | (*b*) For matched studies, give matching criteria and number of exposed and unexposed | NA |
| Variables | 7 | Clearly define all outcomes, exposures, predictors, potential confounders, and effect modifiers. Give diagnostic criteria, if applicable | Exposure (third paragraph of the Methods); Outcome (fifth paragraph of the Methods); potential confounders (sixth paragraph of the Methods). |
| Data sources/ measurement | 8* | For each variable of interest, give sources of data and details of methods of assessment (measurement). Describe comparability of assessment methods if there is more than one group | First, third, fifth and sixth paragraphs of the Methods. |
| Bias | 9 | Describe any efforts to address potential sources of bias | Sixth paragraph of the Methods section. Fourth paragraph of the Statistical analysis section. |
| Study size | 10 | Explain how the study size was arrived at | First paragraph of the Methods section. |
| Quantitative variables | 11 | Explain how quantitative variables were handled in the analyses. If applicable, describe which groupings were chosen and why | First paragraph of the Statistical analysis section. |
| Statistical methods | 12 | (*a*) Describe all statistical methods, including those used to control for confounding | First paragraph of the statistical analysis section. |
|  |  | (*b*) Describe any methods used to examine subgroups and interactions | Second, third and fourth paragraphs of the statistical analysis section. |
|  |  | (*c*) Explain how missing data were addressed | First and fourth paragraphs of the statistical analysis section. |
|  |  | (*d*) If applicable, explain how loss to follow-up was addressed | Third paragraph of the Methods section. |
|  |  | (*e*) Describe any sensitivity analyses | Fourth paragraph of the statistical analysis section. |
| Results | | |  |
| Participants | 13* | (a) Report numbers of individuals at each stage of study—eg numbers potentially eligible, examined for eligibility, confirmed eligible, included in the study, completing follow-up, and analysed | First paragraph of the Results section. |
|  |  | (b) Give reasons for non-participation at each stage | NA |
|  |  | (c) Consider use of a flow diagram | See Figure S1. |
| Descriptive data | 14* | (a) Give characteristics of study participants (eg demographic, clinical, social) and information on exposures and potential confounders | First paragraph of the Results section and Table 1. |
|  |  | (b) Indicate number of participants with missing data for each variable of interest | Table 1. |
|  |  | (c) Summarise follow-up time (eg, average and total amount) | First paragraph of the Results section. |
| Outcome data | 15* | Report numbers of outcome events or summary measures over time | First and second paragraphs of the Results section. |
| Main results | 16 | (*a*) Give unadjusted estimates and, if applicable, confounder-adjusted estimates and their precision (eg, 95% confidence interval). Make clear which confounders were adjusted for and why they were included | Second paragraph of the Results section. |
|  |  | (*b*) Report category boundaries when continuous variables were categorized | NA |
|  |  | (*c*) If relevant, consider translating estimates of relative risk into absolute risk for a meaningful time period | Sixth paragraph of the Results section. |
| Other analyses | 17 | Report other analyses done—eg analyses of subgroups and interactions, and sensitivity analyses | Third, fourth, fifth and seventh paragraphs of the Results section. |
| Discussion | | |  |
| Key results | 18 | Summarise key results with reference to study objectives | First paragraph of the Discussion section. |
| Limitations | 19 | Discuss limitations of the study, taking into account sources of potential bias or imprecision. Discuss both direction and magnitude of any potential bias | Eighth paragraph of the Discussion section. |
| Interpretation | 20 | Give a cautious overall interpretation of results considering objectives, limitations, multiplicity of analyses, results from similar studies, and other relevant evidence | First and final paragraphs of the Discussion section. |
| Generalisability | 21 | Discuss the generalisability (external validity) of the study results | Eighth paragraph of the Discussion section. |
| Other information | | |  |
| Funding | 22 | Give the source of funding and the role of the funders for the present study and, if applicable, for the original study on which the present article is based | Funding section. |

*Give information separately for exposed and unexposed groups.

**Note:** An Explanation and Elaboration article discusses each checklist item and gives methodological background and published examples of transparent reporting. The STROBE checklist is best used in conjunction with this article (freely available on the Web sites of PLoS Medicine at http://www.plosmedicine.org/, Annals of Internal Medicine at http://www.annals.org/, and Epidemiology at http://www.epidem.com/). Information on the STROBE Initiative is available at http://www.strobe-statement.org.
