# Supplementary material for: Preeclampsia and risk of end stage kidney disease: A Swedish nationwide cohort study
Source: PLoS Med. 2019 Jul 30;16(7):e1002875. doi: 10.1371/journal.pmed.1002875 (PMC6667103; doi:10.1371/journal.pmed.1002875)
Supplement: S1 Text — CI, confidence interval; ESKD, end-stage kidney disease; HR, hazard ratio. (DOCX) [file pmed.1002875.s002.docx]

The HRs , 95% CI and p-values for each socio-demographic variable included in the Cox model which was performed to examine the association between pre-eclampsia (in any pregnancy) and the risk of ESKD among healthy women (i.e. no recorded diagnosis of CKD, CVD, diabetes or hypertension before the first pregnancy) are presented in S2 Table. The results suggested that increased BMI (pre-first-pregnancy) was associated with an increased risk of ESKD. The results suggested that the risk of ESKD was increased by 34%, 95% and almost 3-fold among overweight, obese and morbidly obese women respectively, compared to normal BMI women. Non-Scandinavian women were at a 2-fold increased risk of ESKD compared to Swedish women. Women who had high school and university education level had a 40% and 50% reduced risk of ESKD compared to women who had pre-high education level. Women who reported smoking 0-9 or ≥10 cigarettes per day had more than 40% increased risk of ESKD compared to non-smokers. Women who were 40 years old or more at the first pregnancy had an 46% increased risk of ESKD compared to women aged 20-29, however this was not statistically significant.
