# Supplementary material for: Preeclampsia and risk of end stage kidney disease: A Swedish nationwide cohort study
Source: PLoS Med. 2019 Jul 30;16(7):e1002875. doi: 10.1371/journal.pmed.1002875 (PMC6667103; doi:10.1371/journal.pmed.1002875)
Supplement: S2 Text — ESKD, end-stage kidney disease. (DOCX) [file pmed.1002875.s003.docx]

We performed a Cox proportional hazard model to examine the association between pre-eclampsia and end-stage kidney disease (ESKD) among women who had chronic kidney disease (CKD), cardiovascular disease (CVD), hypertension or diabetes. A partially adjusted model was performed adjusting for year of delivery. An adjusted model was performed including socio-demographic factors: maternal age, body mass index, highest education level, smoking and native country, year of delivery and parity. Similar to the main analyses, we repeated the Cox models with stratification by small for gestational age, preterm pre-eclampsia and whether the woman was exposed to pre-eclampsia in one or two pregnancies. The same models were repeated including all women regardless of pre-pregnancy co-morbidity. In addition, we repeated the models while adjusting for the socio-demographic factors and pre-pregnancy CKD, CVD, diabetes and hypertension.

The results of the association between pre-eclampsia and ESKD among women who had been diagnosed with CKD, CVD or diabetes diagnosis before the first pregnancy are reported in in S3 Table. There was a 2-fold increased risk of ESKD in relation to pre-eclampsia (adjusted HR=2.41; [95% CI: 1.79-3.24]), while preterm pre-eclampsia was associated with more than 5-fold increased risk of ESKD and pre-eclampsia and SGA with almost 3-fold increased risk of ESKD.
