# Supplementary material for: Preeclampsia and risk of end stage kidney disease: A Swedish nationwide cohort study
Source: PLoS Med. 2019 Jul 30;16(7):e1002875. doi: 10.1371/journal.pmed.1002875 (PMC6667103; doi:10.1371/journal.pmed.1002875)
Supplement: S3 Text — ESKD, end-stage kidney disease. (DOCX) [file pmed.1002875.s004.docx]

The results of the association between pre-eclampsia and ESKD among all women are presented in S4 Table. There was a 5-fold increased risk of ESKD in relation to pre-eclampsia in the model adjusted for socio-demographic factors (HR=5.67; [95% CI: 4.70-6.84]) which was reduced to (HR=4.25; [95% CI: 3.50-5.16]) when the model was adjusted for pre-pregnancy co-morbidity. More detailed results highlighting the confounding effect of pre-pregnancy co-morbidity are in S4 Table.
