# Supplementary material for: Preeclampsia and risk of end stage kidney disease: A Swedish nationwide cohort study
Source: PLoS Med. 2019 Jul 30;16(7):e1002875. doi: 10.1371/journal.pmed.1002875 (PMC6667103; doi:10.1371/journal.pmed.1002875)
Supplement: S4 Text — ESKD, end-stage kidney disease. (DOCX) [file pmed.1002875.s005.docx]

To estimate the risk of ESKD at five, 10 and 20 years post the first delivery in relation to pre-eclampsia, the Cox regression models described in the statistical analysis section were repeated with follow-up stopped at five years, 10 years and 20 years, respectively. We performed the models for 1) healthy women; 2) women with co-morbidity before the first pregnancy (CKD, CVD, diabetes or hypertension); and 3) all women regardless of pre-pregnancy co-morbidity (S5 Table). The variables included in each model are listed in the Table footnote. Among healthy women, the highest HRs were found at five and 10 year follow-up (HR range between 7 and 8 in the crude and adjusted models), while the HR at 20 year follow-up was five-fold. Among women with pre-pregnancy co-morbidity, the HRs were two-fold in all analyses. Among all women, the HRs were between six and seven in the crude models and the models adjusted for socio-demographic factors, and decreases to between three and four when pre-pregnancy co-morbidities were adjusted for.
